# Editorial Comment: Practice Patterns and Impact of Postchemotherapy Retroperitoneal Lymph Node Dissection on Testicular Cancer Outcomes

**DOI:** 10.1590/S1677-5538.IBJU.2020.04.08

**Published:** 2020-05-20

**Authors:** Gustavo Cardoso Guimarães

**Affiliations:** 1 Departamento de Oncologia Cirúrgica Beneficência Portuguesa de São Paulo São PauloSP Brasil Chefe do Departamento de Oncologia Cirúrgica, Beneficência Portuguesa de São Paulo, São Paulo, SP, Brasil

Woldu SL #^1^, Moore JA #^2^, Ci B ^3^, Freifeld Y ^1^, Clinton TN ^1^, Aydin AM ^4^, et al.

# Contributed equally

^1^ Department of Urology, University of Texas Southwestern Medical Center, Dallas, TX, USA;^2^ Department of Medicine, Division of Hematology/Oncology, University of Texas Southwestern Medical Center, Dallas, TX, USA;^3^ Department of Clinical Science, Division of Biomedical Informatics, University of Texas Southwestern Medical Center, Dallas, TX, USA;^4^ Department of Urology, Hacettepe University, Ankara, Turkey

Eur Urol Oncol. 2018 Aug;1(3):242-251

**DOI: 10.1016/j.euo.2018.04.005 | ACCESS: 10.1016/j.euo.2018.04.005**

## COMMENT

In this paper, Dr. Solomon L. Woldu, and colleagues, from University of Texas Southwestern Medical Center, Dallas, TX, USA, evaluated patterns of postchemotherapy retroperitoneal lymph node dissection (PC-RPLND) use in the USA and evaluate the association between PC-RPLND and survival in advanced nonseminomatous germ cell tumors (NSGCTs).

They conduct a retrospective, observational study using National Cancer Data Base (NCDB) data from 2004–2014 for 5062 men diagnosed with stage II/III NSGCT.

PC-RPLND plays a central role in the multidisciplinary approach of patients with advanced testicular cancer, removing lymph nodes that may contain viable tumor or teratoma, with prognostic implications and impact on survival and

30% of patients after chemotherapy with visible persistent masses on examination and negative serum tumor markers are eligible for PC-RPLND because these masses can harbor viable GCT or teratoma.

The authors find that patients undergoing PC-RPLND were more likely to be younger, white, privately insured, and reside in more educated/wealthier regions (p < 0.001). Insurance status was independently associated with receipt of PC-RPLND; compared to patients with private insurance, those without insurance were significantly less likely to receive PC-RPLND.

After multivariate adjustment, age, comorbidity, non-private insurance, distance from hospital, clinical stage, and risk group were independently associated with all-cause mortality and omission of PC-RPLND remained associated with all-cause mortality (hazard ratio 1.98; p < 0.001).

These data reinforce the need to subject these patients to, because omission of PC-RPLND is associated with lower OS.

